# Graphene Architecture-Supported Porous Cobalt–Iron Fluoride Nanosheets for Promoting the Oxygen Evolution Reaction

**DOI:** 10.3390/nano14010016

**Published:** 2023-12-20

**Authors:** Yanhui Lu, Xu Han, Yiting Zhang, Xu Yu

**Affiliations:** School of Chemistry and Chemical Engineering, Yangzhou University, Yangzhou 225000, China221004237@stu.yzu.edu.cn (Y.Z.)

**Keywords:** fluoridation, OER, hierarchical structure, graphene

## Abstract

The design of efficient oxygen evolution reaction (OER) electrocatalysts is of great significance for improving the energy efficiency of water electrolysis for hydrogen production. In this work, low-temperature fluorination and the introduction of a conductive substrate strategy greatly improve the OER performance in alkaline solutions. Cobalt–iron fluoride nanosheets supported on reduced graphene architectures are constructed by a one-step solvothermal method and further low-temperature fluorination treatment. The conductive graphene architectures can increase the conductivity of catalysts, and the transition metal ions act as electron acceptors to reduce the Fermi level of graphene, resulting in a low OER overpotential. The surface of the catalyst becomes porous and rough after fluorination, which can expose more active sites and improve the OER performance. Finally, the catalyst exhibits excellent catalytic performance in 1 M KOH, and the overpotential is 245 mV with a Tafel slope of 90 mV dec^−1^, which is better than the commercially available IrO_2_ catalyst. The good stability of the catalyst is confirmed with a chronoamperometry (CA) test and the change in surface chemistry is elucidated by comparing the XPS before and after the CA test. This work provides a new strategy to construct transition metal fluoride-based materials for boosted OER catalysts.

## 1. Introduction

With the rapid development of society and the increasing energy demand, pollution caused by the combustion of fossil fuels is an emerging issue to be solved [1,2,3,4]. Electrocatalytic water splitting is a promising option to produce renewable hydrogen energy and decrease environmental pollution. In practical water splitting, it is impossible to directly produce hydrogen because the efficiency of the reaction is very low. Functionalized catalysts not only reduce the activation energy of the electrolyzed water reaction but also decrease the overpotential during the oxygen evolution reaction (OER) [5,6]. Therefore, the quality of the catalyst determines the total voltage for the practical electrolysis of water and the conversion efficiency of electrical energy into hydrogen energy. In alkaline electrolytes, noble metals such as platinum (Pt) and ruthenium (Ru) oxides are still good catalysts in terms of catalytic effects, but the scarcity of their sources still hinders their practical application for producing hydrogen.

Many efforts to develop low-price and effective catalysts have been carried out [7]. Transition metal (TM)-based nanomaterials, including transition metals [8,9,10], metal alloys [11,12], perovskites [13], metal oxides/sulfides [12,14], etc., have attracted attention due to their variable electronic state distribution, rich species, and tunable structure to maintain good electrocatalytic performance [15]. Heteroatom (e.g., sulfur and phosphorus)-incorporated TM compounds can show enhanced electrocatalytic OER performance attributed to adjusted surface chemistry and the formation of a covalent bond [16,17,18,19], such as CoS with a hexagonal bipyramid structure [18], amorphous–crystalline CoS/Ni_x_P_y_/Fe-Ni_3_S_2_ [20], CoS/Co_3_O_4_ nanoframes [19], etc. Fluorine has the largest electronegativity, and fluorine incorporation into TM compounds has been proven to show excellent OER activity due to the co-existence of ionic metal-F and metal-O bonds [10,21,22], such as fluoridated NiFe layered double hydroxides [21] and porous CoFe-F nanocubes [22]. However, the poor conductivity of TM-based catalysts can result in a decrease in electrochemical performance due to the existence of unstable oxygen content. Meanwhile, the catalytic reaction occurs at the surface or subsurface, and the limited degree of self-reconfiguration can result in a decreased number of active sites in the near-surface region and leave a large number of inactive atoms in the bulk, which is not conducive to improving the catalytic activity [23,24]. To improve the conductivity of transition metal-based catalysts and expose more active sites, the most promising strategies are as follows: (1) Introducing new compounds to synthesize polymetallic compounds and use their synergistic effects to tune electronic structures [25]; (2) constructing porous structures, increasing their surface area, and exposing more active sites [26]; (3) introducing a conductive substrate to improve the charge transfer ability of the catalyst [23], such as carbon nanotubes [27,28] and graphene [29,30,31]. Graphene as a layered nanomaterial can be further introduced to construct a hierarchical structure via its self-assembly of functionalized nanosheets, which can accelerate ion diffusion and charge transfer and improve the conductivity of compounds [32]. The strong coupling of hierarchical graphene with transition metal fluorides not only provides abundant channels but also inhibits the aggregation and stacking of transition metals, exposing more active sites to realize an enhancement in the catalytic performance [33,34].

Here, we prepared cobalt–iron fluoride nanosheets supported on reduced graphene architectures (CoFeF-GA) via the precipitation, one-step hydrothermal, and fluoridation methods. The hierarchical structure can expose more active sites and the formation of ionic metal-F bonds is favorable for adjusting the surface chemistry of catalysts. The required overpotential of CoFeF-GA in 1 M KOH solution is 245 mV at 10 mA cm^−2^, and the Tafel slope of the material is 90 mV dec^−1^, which is superior to commercial IrO_2_ catalysts. This work provides a new concept to prepare transition metal-based materials with improved OER activity and stability for energy applications.

## 2. Materials and Methods

### 2.1. Chemicals

All chemicals were purchased and used without further purification. Ammonium fluoride (NH_4_F), cobalt nitrate hexahydrate (Co(NO_3_)_2_·6H_2_O), iron nitrate nonahydrate (Fe(NO_3_)_3_·9H_2_O), ethylene glycol, and ammonium hydroxide were purchased from Shanghai Aladdin Bio-Chem Technology Co., Ltd. (Shanghai, China). The graphene oxides were purchased from Global Graphene Group. Sodium carbonate and potassium hydroxide (KOH AR) were purchased from Sinopharm Chemical Reagent Co., Ltd. (Shanghai, China). Ultrapure water was used throughout the experiments.

### 2.2. Synthesis of CoFeF

Firstly, 12.5 mL of ammonium hydroxide (NH_3_·H_2_O) was mixed with 15 mL of ethylene glycol under stirring for 2 min. Secondly, 3.5 mL of 1 M Na_2_CO_3_, 4 mL of 1 M Co(NO_3_)_2,_ and 1 mL of 1 M Fe(NO_3_)_3_ were added to the mixture while stirring for 20 min. Then, the final mixture was transferred to a 50 mL Teflon-lined high-temperature autoclave and heated at 170 °C for 17 h. After cooling down to room temperature, the products were washed with ethanol/deionized water several times and dried at 60 °C overnight under vacuum conditions. The as-obtained powder and ammonium fluoride (NH_4_F) were placed at the downstream and upstream sides of a porcelain boat. The porcelain boat was fixed in the center of a tube furnace. The mass ratio of NH_4_F to the CoFe precursor is 16:1. Annealing was carried out at 320 °C for 2 h in a N_2_ atmosphere, and the heating rate was 3 °C min^−1^ with a N_2_ flow of 10 cc min^−1^. Finally, the target material was obtained and named CoFeF. In comparison, the control sample was prepared using the same method without the fluoridation and named CoFe.

### 2.3. Synthesis of CoFeF-GA

Firstly, 12.5 mL of ammonium hydroxide (NH_3_·H_2_O) was mixed with 15 mL of ethylene glycol under stirring for 2 min. Secondly, 3.5 mL of 1 M Na_2_CO_3_, 4 mL of 1M Co(NO_3_)_2_, 1 mL of 1 M Fe(NO_3_)_3,_ and 50 mg of graphene oxides were added to the mixture while stirring for 20 min. Then, the final mixture with a violet color was transferred to a 50 mL Teflon-lined high-temperature autoclave and heated at 170 °C for 17 h. After cooling down to room temperature, the products were washed with ethanol/deionized water several times and freeze-dried under vacuum conditions. After that, the as-obtained CoFe-GO precursor and ammonium fluoride (NH_4_F) were placed at the downstream and upstream sides of a porcelain boat. The porcelain boat was put in the center of a tube furnace. The mass ratio of NH_4_F to CoFe-GO precursor is 16:1. The annealing temperature was kept at 320 °C for 2 h in a N_2_ atmosphere and the heating rate was 3 °C min^−1^ with a N_2_ flow of 10 cc min^−1^. Finally, the target material was obtained and named CoFeF-GA. In comparison, the control sample was prepared using the same method without the fluoridation and named CoFe-GA.

### 2.4. Characterizations

All samples were analyzed using a scanning electron microscope (SEM, Hitachi, S-4800 II, Tokyo, Japan), transmission electron microscope (TEM), high-resolution transmission electron microscope (HRTEM, Philips, TECNAI 12, Amsterdam, The Netherlands), and the related element mapping analysis (200 kV Philips TECNAI G2 electron microscope). The KEVEX X-ray energy detector was used for energy color scattered X-ray (EDS) analysis. Powder X-ray diffraction (XRD) patterns were recorded on a Bruker D8 Advance powder using a Cu Kα (λ = 1.5405 Å) radiation source, operating at 40 kV and 40 mA at a scanning rate of 5° min^−1^. Surface analysis of the sample was studied by X-ray photoelectron spectroscopy (XPS, Thermo Science, ESCALAB 250Xi, Waltham, MA, USA). Thermogravimetric analysis (TGA) was carried out on a NETZSCH TG 209 F3 with a heating rate of 10 °C min^−1^ from room temperature to 600 °C under a N_2_ atmosphere.

### 2.5. Electrochemical Measurements

The electrochemical measurements were performed in a 1.0 M KOH electrolyte at room temperature using an electrochemical workstation (CHI660E, Shanghai, China) with a three-electrode system. A glassy carbon electrode (GC, diameter of 3 mm, 0.07 cm^2^) was used as the supporting working electrode. All potentials were referenced to a reversible hydrogen electrode (RHE); the formula is as follows: E_(RHE)_ = E_(SCE)_ + 0.059 × pH + 0.242V. A graphite rod and saturated calomel electrode (SCE) were used as the counter and reference electrodes, respectively. The uniform catalyst ink was prepared as follows: 5 mg of catalyst, 950 μL of ethanol, and 50 μL of 5 wt% Nafion were mixed and ultrasonicated for 1 h. Then, 10 μL of catalyst ink was loaded dropwise on to the glassy carbon electrode under natural drying, and then the as-prepared working electrode was immersed in the electrolyte. The mass loading of the catalyst supported on the glassy carbon electrode was about 0.71 mg cm^−2^, and the SCE was calibrated before and after the electrochemical test. The potential was calibrated to the reversible hydrogen electrode (RHE) using the equation: E_RHE_ = E_SCE_ + 0.059pH + 0.241 − IR. E_SCE_ is the experimental potential measured against the SCE reference electrode, and 0.241 is the standard potential of the SCE at 25 °C. The equation of η(V) = E_(RHE)_ − E_θ_ was used to calculate the overpotential of these electrocatalysts, where E_θ_ represents the thermodynamic potential for OER (1.23 V vs. RHE).

The working electrode was pre-treated in 1M KOH with a N_2_ flow before the electrochemical test. The polarization curves were measured by cyclic voltammetry (CV) at a scan rate of 5 mV s^−1^. The ohmic resistance with IR correction was obtained using electrochemical impedance spectroscopy in the frequency range of 1000 kHz~10 mHz with an amplitude of 5 mV. A durability test was carried out by CV for 1000 cycles with the potential ranging from 1.05 to 1.55 V vs. RHE in 1 M KOH at 150 mV s^−1^, and a linear sweep was measured under a scan rate of 5 mV s^−1^ for 1000 cycles. Chronoamperometry (CA) was measured for 10 h. The electrochemical surface area (ECSA) was evaluated based on the double-layer capacitance (C*_dl_*), and the ECSA value was estimated by CV without Faradaic processes occurring in the region. CV curves were measured at scan rates from 20 to 100 mV s^−1^ and the applied potential was from 1.02 V to 1.12 V.

The Faradaic efficiency of CoFe-F-16 was measured at 1.48 V for 1 h and calculated using the following equation.

$$Faradaic\;yield=\frac{V_{\mathrm{Exp}}}{V_{\mathrm{Theor}}}=\frac{V_{\mathrm{Exp}\;}}{\frac{1}{4}\times \frac{Q}{F}\times V_{m}}$$

where V_Exp_ and V_Theor_ are the experimental and theoretic volumes of the generated O_2_ gas during the catalytic process, Q is the charge passed through the electrode, *F* is the Faraday constant (96485 C mol^−1^), the number 4 means 4-mole electrons per mole of O_2_, and V_m_ is the molar volume of gas (24.5 L mol^−1^, 298 K, 101 KPa). 

## 3. Results

### 3.1. The Morphological Structure of CoFeF-GA

**Figure 1:**
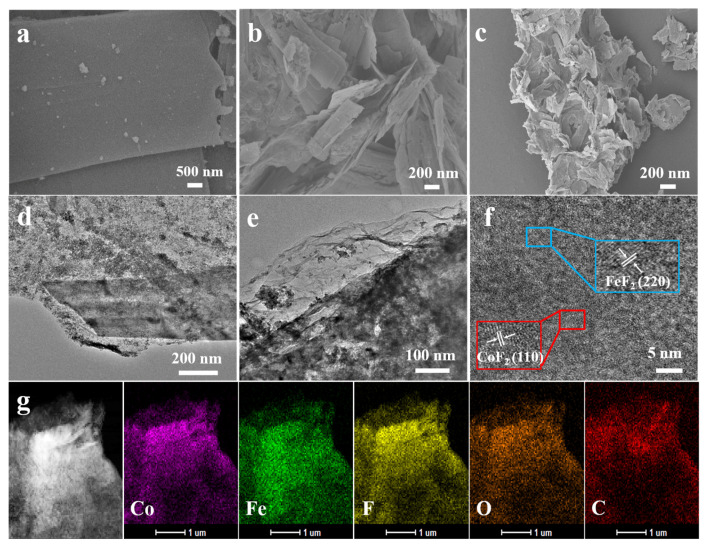
(**a**) Low-magnification SEM image of CoFe hydroxy-carbonate precursor. High-magnification SEM images of (**b**) CoFe-GA and (**c**) CoFeF-GA. (**d**,**e**) TEM images of the CoFeF-GA. (**f**) High-resolution TEM image of CoFeF-GA. (**g**) STEM image and the corresponding elemental mappings of CoFeF-GA (elements: Co, Fe, F, O, and C).

The catalyst consisting of cobalt–iron fluoride nanosheets supported on reduced graphene architectures (CoFeF-GA) was prepared by a hydrothermal method and low-temperature fluorination. Briefly, iron nitrate, cobalt nitrate, and graphene oxides were mixed in ammonium hydroxide and ethylene glycol, and sodium carbonate was used to adjust the pH. During the hydrothermal process, the hierarchical structure was constructed by the self-assembly of graphene nanosheets, which were well-maintained by the sublimation of solvent after the freeze-drying treatment. Finally, the hierarchical CoFeF-GA was obtained and graphene oxides were reduced during the further fluoridation treatment. To investigate the morphological structure, scanning electron microscopy (SEM) was initially carried out, as shown in Figure 1a–c. In comparison to the pristine CoFe nanosheets (Figure 1a), a small and thin layer of nanosheets can be observed for CoFe-GA in Figure 1b. After fluoridation treatment, CoFeF-GA showed a hierarchical structure and rough surface (Figure 1c), which allowed the exposure of a large number of active sites. The nitrogen adsorption/desorption analysis is evaluated in Figure S1, and the specific surface area of CoFeF-GA was about 135 cm^2^ g^−1^, with an average diameter of 22.4 nm and pore volume of 4.32 cm^3^ g^−1^. Coupling reduced graphene oxides with CoFeF nanosheets and the formation of ionic metal-F bonds are favorable to improving the conductivity of CoFeF-GA. 

To further verify the morphology of CoFeF-GA, transmission electron microscopy (TEM) and high-resolution transmission electron microscopy (HR-TEM) were conducted. As shown in Figure 1d,e, CoFeF nanosheets are deposited on the conductive graphene surface, and the rough surface of CoFeF-GA is caused by the shrinkage of graphene nanosheets and fluorine incorporation into the CoFe hydroxy-carbonate. Figure 1f shows the HR-TEM image of CoFeF-GA, and the fringed lattice spacing of 0.33 and 0.37 nm corresponds to the (110) crystal plane of CoF_2_ and (220) crystal plane of FeF_2_, respectively. The elemental mapping images of the CoFeF-GA catalyst imply a uniform distribution of C, O, Co, Fe, and F elements (Figure 1g), which was further confirmed by energy-dispersive X-ray spectroscopy (EDS) (Figure S2). The oxygen species are possibly from the partial surface oxidation or the surface adsorption of oxygen, and the uniform distribution of the F element indicates successful fluorination treatment.

**Figure 2:**
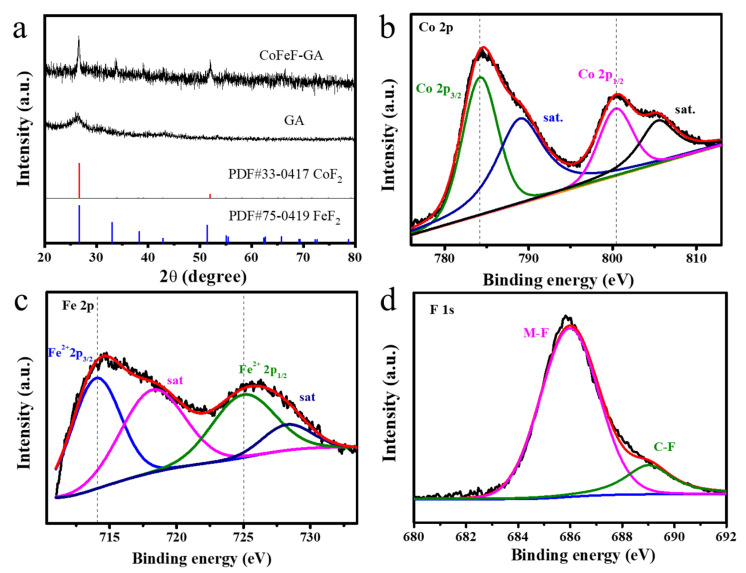
(**a**) XRD patterns of GA and CoFeF-GA. The high-resolution XPS spectra of (**b**) Co 2p (**c**) Fe 2p and (**d**) F 1s for CoFeF-GA.

The X-ray diffraction (XRD) technique was carried out to characterize the crystal structure of CoFeF-GA. As shown in Figure 2a, the CoFeF-GA catalyst has typical diffraction peaks corresponding to a hexagonal crystal structure. The diffraction peaks at 26.73°, 33.08°, and 51.45° correspond to the (110), (101), and (211) planes of FeF_2_ (PDF #75-0419), and the diffraction peaks at 26.73° and 52.07° are index to the (110) and (211) planes of CoF_2_ (PDF #33-0417), respectively. The main peak of graphene overlaps with the diffraction peaks of CoF_2_ and FeF_2_, and no additional diffraction peaks can be found, indicating the successful fluorination of the CoFeF-GA catalyst. To determine the proportion of graphene oxide and metal compounds for CoFeF-GA, thermogravimetric analysis (TGA) was carried out, as shown in Figure S3. The initial weight loss of 16.8% near 120 °C corresponds to the removal of impurities and solvents, and the weight loss from 120 to 450 °C is attributed to a reduction in graphene oxides. Finally, the conversion from metal fluorides to metal oxides occurs above a temperature of 450 °C and the final weight loss is about 37.3%.

The surface chemical valence of CoFeF-GA was studied by X-ray photoelectron spectroscopy (XPS). All the peaks were calibrated by C 1s peak at 284.6 eV. The full XPS spectrum of the CoFeF-GA catalyst shows Fe, Co, F, and C peaks at 715, 783, 685, and 284 eV (Figure S4), which is well matched with the EDS and elemental mapping results. The high-resolution Co 2p spectrum (Figure 2b) shows the primary peaks of Co^2+^ [35], and the divided peaks at 784.1 and 801.2 eV are attributed to the spin−orbits of Co 2p_3/2_ and Co 2p_1/2_ accompanying the satellite peaks. For the Fe 2p spectrum in Figure 2c, the fitted peaks at 713.6 and 725.1 eV correspond to the spin–orbits of Fe 2p_3/2_ and Fe 2p_3/2_ for Fe^2+^ [5], and the peaks at 716.8 eV and 728.3 eV are indexed to the related satellite peaks. Figure 2d shows two peaks at 686.2 eV and 689.2 eV corresponding to the metal-F and C-F bonds, respectively, which demonstrate the successful fluoridation of CoFeF-GA. 

### 3.2. Electrochemical Performance

**Figure 3:**
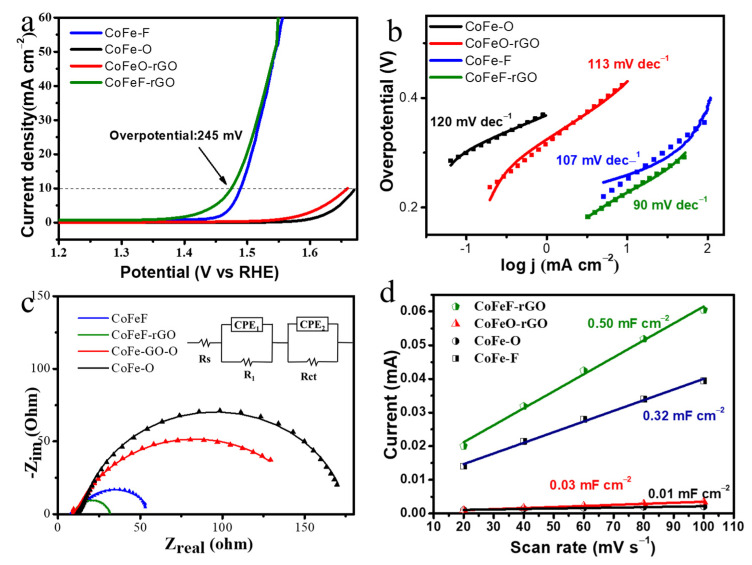
Electrocatalytic OER performance in 1 M KOH solution. (**a**) The OER polarization curves and (**b**) Tafel plots of CoFeF-GA, CoFe-GA, CoFe, CoFeF. (**c**) Nyquist plots of CoFeF-GA, CoFe-GA, CoFe, CoFeF (inset: the electrical equivalent circuit). (**d**) C_dl_ values of CoFeF-GA, CoFe-GA, CoFe, CoFeF.

The electrochemical performance of CoFeF-GA was measured using a three-electrode configuration in 1 M KOH with nitrogen purification. The electrocatalytic OER activity of CoFeF-GA, CoFe-GA, CoFe, and CoFeF was initially evaluated via a linear sweep voltammetry (LSV) measurement at a scan rate of 5 mV s^−1^. As shown in Figure 3a, CoFeF-GA afforded an overpotential of 245 mV to reach a current density of 10 mA cm^−2^, which was lower than that of CoFe-F (252 mV), CoFe-GA (420 mV), and CoFe (440 mV) and other reported transition metal catalysts (Table S1), respectively. The slow kinetics of oxygen desorption of oxides led to poor OER performance for CoFe and CoFe-GA. After the fluorination treatment, the required overpotential for CoFeF was 252 mV at 10 mA cm^−2^, and the catalytic performance was significantly enhanced, which proves that the heteroatom fluorine doping strategy with the formation of an ionic metal-F bond has a positive effect on the improvement in the catalytic performance. Furthermore, a lower overpotential for CoFeF-GA than CoFeF can be attributed to the good electrical conductivity and large surface area due to the introduction of the conductive graphene, which can expose more active sites and provide fast ion transfer channels. 

The Tafel slope is an important parameter to reveal the reaction mechanism. As shown in Figure 3b, the Tafel slope of CoFeF-GA is 90 mV dec^−1^, which is smaller than that of CoFeF (107 mV dec^−1^), CoFe-GA (113 mV dec^−1^), and CoFe (120 mV dec^−1^). A smaller value of the Tafel slope implies a faster increase in current density and a smaller change in overpotential (η). This result confirms that CoFeF-GA has rapid kinetic behavior and good electrocatalytic OER performance. The surface dynamic performance and electrode interface properties were studied by electrochemical impedance spectroscopy (EIS). Figure 3c shows the Nyquist plots of all samples, which are fitted according to the electrical equivalent circuit (inset of Figure 3c). The related resistances R_s_, R_1,_ and R_ct_ represent the solution resistance of the electrolyte, the catalyst membrane resistance, and the charge transfer resistance. At high frequency, the value of R1 is recorded at the highly porous surface and the reaction on the catalyst. CoFeF-GA shows a smaller semicircle than other samples, implying a faster charge transfer rate during the OER process, which is in agreement with the result of Tafel slopes. The fitted Nyquist plots are listed in Table S2. The double-layer capacitance (C_dl_) was calculated by cyclic voltammetry (CV) in the non-faradic region from 20 to 100 mV s^−1^ (Figure S5), and electrochemical surface area (ECSA) is proportional to C_dl_, which can be effectively calculated from the values of C_dl_. As shown in Figure 3d, the C_dl_ of CoFeF-GA (0.5 mF cm^−2^) is higher than that of CoFeF (0.32 mF cm^−2^), CoFe-GA (0.03 mF cm^−2^), and CoFe (0.01 mF cm^−2^) in Table S3, indicating a larger active surface for CoFeF-GA. The large values of ECSA, C_dl,_ and R_f_ predict that CoFeF-GA can provide sufficient open space and surface area for fast electrolyte diffusion and charge transfer. 

**Figure 4:**
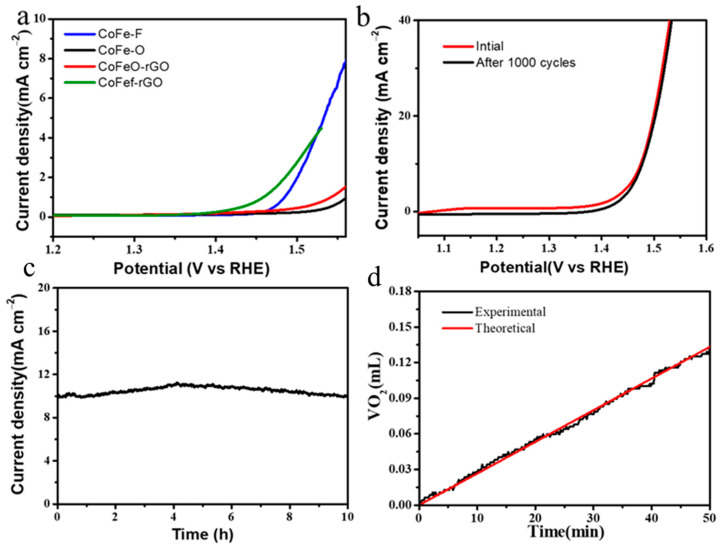
(**a**) Specific activity of CoFeF-GA, CoFe-GA, CoFe, and CoFeF in 1 M KOH solution. (**b**) CV curves before and after 1000 cycles of CoFeF-GA, (**c**) chronoamperometry measurement of CoFeF-GA at a potential of 1.475 V, and (**d**) current efficiency of CoFeF-GA for OER during potential electrolysis at 1.475 V in 1 M KOH.

The specific activity was compared by normalizing the raw current to the ECSA. At an overpotential of 300 mV, the specific activity of CoFeF-GA was 4.7, which was about 5.8 and 11.8 times that of CoFe-GA and CoFe (Figure 4a). A similar specific activity for CoFeF-GA and CoFeF is attributed to the formation of metal-F bonds after the fluoride element incorporation. To confirm the catalytic stability, the CV curves for 1000 cycles and chronoamperometry (CA) were measured. After 1000 cycles, the CV curve almost overlaps with its initial cycle, and the change in overpotential is almost ignored (Figure 4b), indicating a high catalytic OER stability for CoFeF-GA as the anode catalyst in an alkaline electrolyte. Furthermore, the durability was further confirmed by the CA test at 1.475 V, and the current density was slightly changed after the CA test for 10 h (Figure 4c). Faraday efficiency is an important indicator for the practical application of catalysts, which is determined by matching the generated experimental amount of oxygen with the theoretical amount of oxygen. After the faradic efficiency was measured at a potential of 1.475 V for 50 min, the calculated current efficiency for CoFeF-GA was close to 100% (Figure 4d), indicating good electrocatalytic stability.

**Figure 5:**
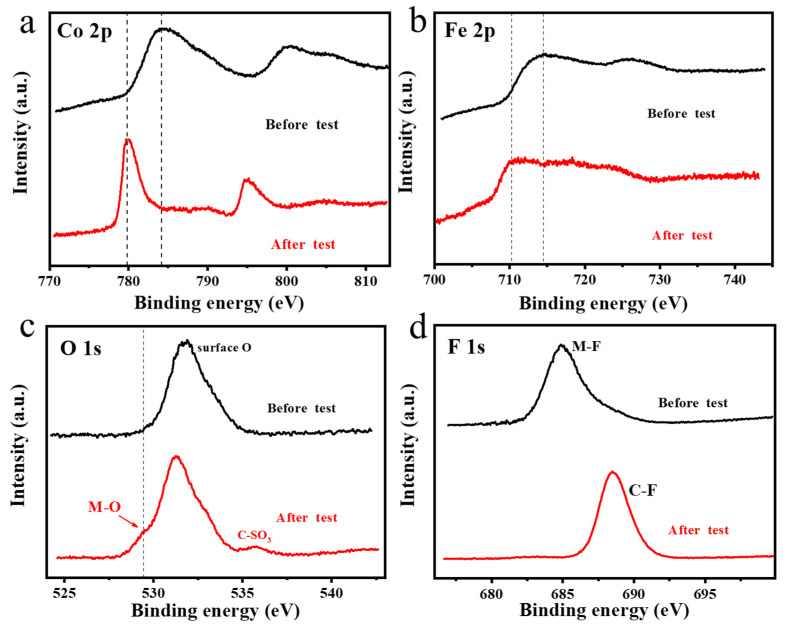
High-resolution XPS spectrum of (**a**) Co 2p, (**b**) Fe 2p, (**c**) O 1s, and (**d**) F 1s for CoFeF-GA before and after stability test.

The morphological and crystal structure of CoFeF-GA after the stability test were probed by TEM and XRD. It was found that the surface of CoFeF-GA after the stability test became dark and the typical characteristic peaks of metal fluorides disappeared, resulting from the formation of metal oxyhydroxides during the OER test (Figure S6). To investigate the change in the surface chemistry of CoFeF-GA, XPS spectra before and after the stability test were compared. It can be observed that the Co 2p and Fe 2p spectra of CoFeF-GA after the CA test shifted to a lower binding energy than the pristine state (Figure 5a,b). The disappearance of the metal-F bond and the formation of the metal-O bond were assigned to the surface oxidation of CoFeF-GA during the OER process. Since the electronegativity of fluorine is greater than that of oxygen, the electrons are more easily detached from the metal sites, resulting in the shift of the primary peak. As further confirmed by the O 1s spectra in Figure 5c, the metal-O bond appears at 529.8 eV after the CA test, and the peak at 535.4 eV corresponds to the C-SO_3_ arising from the Nafion as the binder. For F 1s spectra in Figure 5d, the disappearance of the metal-F bond results from the formation of the metal-O bond at high potential by surface oxidation during the OER process. The above results indicate that the surface of OER catalysts will self-restructure to form oxides or hydroxyls at high anode potentials, and the new phases formed are the real catalytic active sites [36].

The active phase of CoFeF-rGA is a high-valence state of MOOH driven by the high potential under alkaline conditions. According to the reported catalytic mechanism, the basic principle of the catalytic OER for CoFeF-rGA is presented as follows. In the catalytic process, the M-F bond will be dissociated to form M-OH, which will be finally transferred to M-O and M-OOH species at a high oxidation potential [37]. The critical intermediates of MOO^−^ as the active oxygen species convert to O_2_ gas, which can provide a new position for the next cycle. The most likely reaction mechanisms are listed as Equations 1 to 4 [38,39].

M + OH^−^ → M-OH + e^−^ (1)

M-OH + OH^−^ → M-O + H_2_O + e^−^ (2)

M-O + OH^−^ → M-OOH + e^−^ (3)

M-OOH + OH^−^ → M + H_2_O + O_2_ + e^−^ (4)

As demonstrated by the above results, CoFeF-GA shows excellent electrocatalytic OER activity, which can be attributed to the following possibilities. (1) The hierarchical structure and rough surface of the catalyst can guarantee the exposure of a large active surface, provide many ion diffusion pathways, and improve the utilization of active sites; (2) the formation of the ionic metal-F bond by heteroatom fluorine doping can adjust the surface chemistry of the catalyst to boost the surface active sites; (3) the introduction of conductive graphene can increase the electrical conductivity and prevent the aggregation of CoFeF nanosheets during the synthesis process to develop the electrocatalytic stability of CoFeF-GA; (4) CoFeF coupled with reduced graphene oxides can boost the active sites and reduce the reaction barrier of the catalyst to develop its electrocatalytic activity for the OER in an alkaline electrolyte. 

## 4. Conclusions

In this work, a hierarchical and effective CoFeF-GA catalyst was constructed by the hydrothermal and fluoridation methods. The hierarchical structure of CoFeF-GA was formed by the crosslink of graphene nanosheets via a π-π configuration, which can guarantee the exposure of abundant active sites and provide many pathways for fast ion diffusion. Meanwhile, the surface chemistry was adjusted by heteroatom fluorine incorporation, and the formation of ionic metal-F bonds was favorable for boosting the active sites, improving the electrocatalytic OER performance. Due to the structural and chemical effects, CoFeF-GA shows excellent OER performance in alkaline electrolytes, such as the low overpotential of 245 mV to afford a current density of 10 mA cm^−2^ and its small Tafel slope of 90 mV dec^−1^. The low R_ct_ value of CoFeF-GA implies fast dynamic behavior during the OER process. This work provides a facile strategy to prepare hierarchical transition metal fluoride-based catalysts for energy conversion systems.

## Data Availability

The data presented in this study are available on request from the corresponding author.

## Supplementary Materials

The following supporting information can be downloaded at: https://www.mdpi.com/article/10.3390/nano14010016/s1, Figure S1: The nitrogen adsorption/desorption isotherms of CoFeF-GA. Figure S2: Energy dispersive X-ray spectroscopy of CoFeF-GA. Figure S3: TGA full spectrum of the CoFeF-GA. Figure S4: XPS full spectrum of the CoFeF-GA. Figure S5: CV curves of (a) CoFe, (b) CoFe-GA, (c) CoFeF (d) CoFeF-GA in 1 M KOH at scan rates of 20, 40, 60, 80 and 100 mV s^−1^. Figure S6: TEM image and XRD pattern of the CoFeF-GA catalyst after continuous CA test in 1.0 M KOH. Table S1: The comparison of other OER catalysts derived from transition metal-based materials in 1M KOH (η: overpotential at the current density of 10 mA cm^−2^). Table S2. The fitted Rs, Rct and R1 values of all catalysts. Table S3. Detailed values of Cdl, ECSA and Rf of all catalysts. References [40,41,42,43,44,45,46,47,48,49,50] are cited in the Supplementary Materials.
