# Supplementary material for: Graphene Architecture-Supported Porous Cobalt–Iron Fluoride Nanosheets for Promoting the Oxygen Evolution Reaction
Source: Nanomaterials (Basel). 2023 Dec 20;14(1):16. doi: 10.3390/nano14010016 (PMC10780600; doi:10.3390/nano14010016)
Supplement: Supplementary file 1 [file nanomaterials-14-00016-s001.zip › nanomaterials-2747760-supplementary.pdf]

Supporting information

Graphene Architecture-Supported Porous Cobalt–Iron Fluoride Nanosheets for Promoting the Oxygen Evolution Reaction

Yanhui Lu, Xu Han, Yiting Zhang and Xu Yu *

School of Chemistry and Chemical Engineering, Yangzhou University, Yangzhou 225000, China; 221004237@stu.yzu.edu.cn (Y.Z.)

* Correspondence: yxypz15@yzu.edu.cn

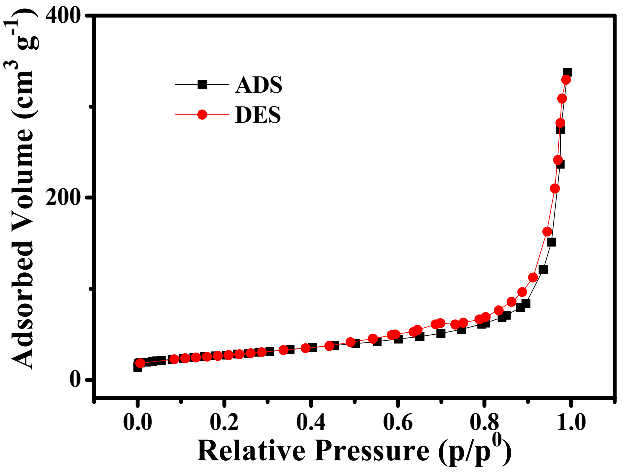

Figure S1 The nitrogen adsorption/desorption isotherms of CoFeF-GA.

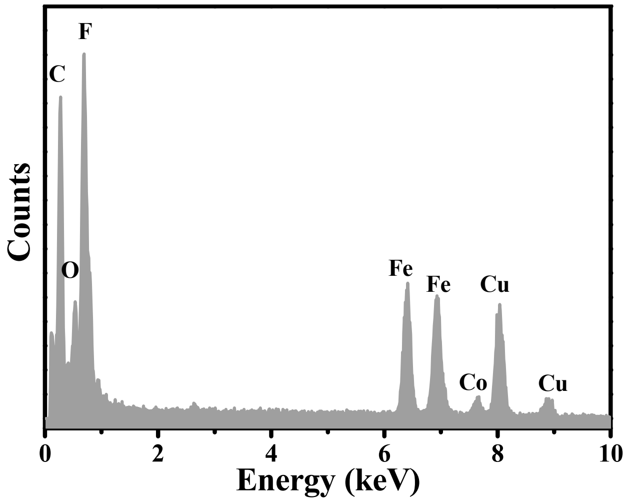

Figure S2 Energy dispersive X-ray spectroscopy of CoFeF-GA.

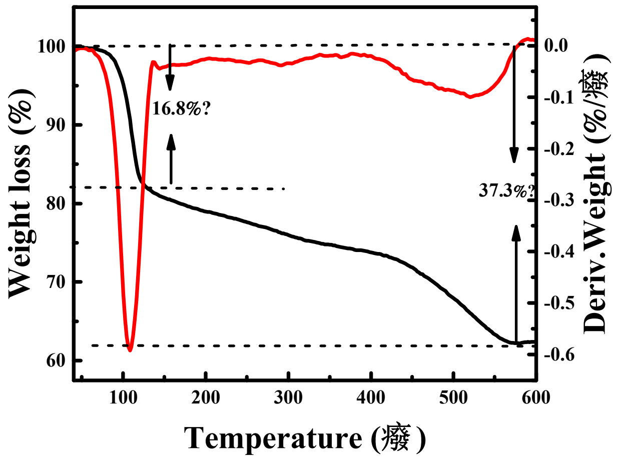

Figure S3 TGA full spectrum of the CoFeF-GA

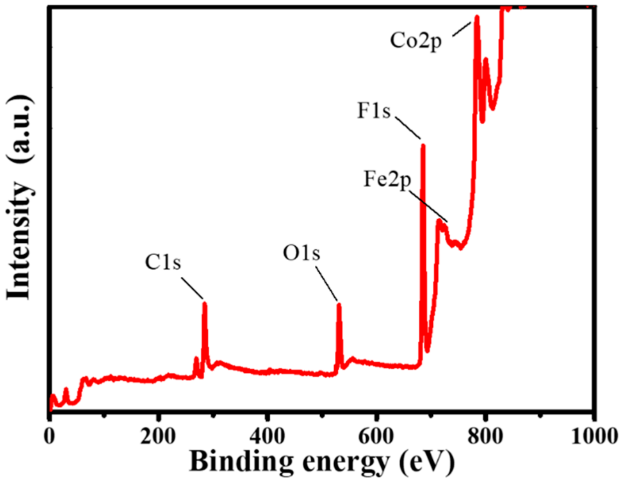

Figure S4 XPS full spectrum of the CoFeF-GA.

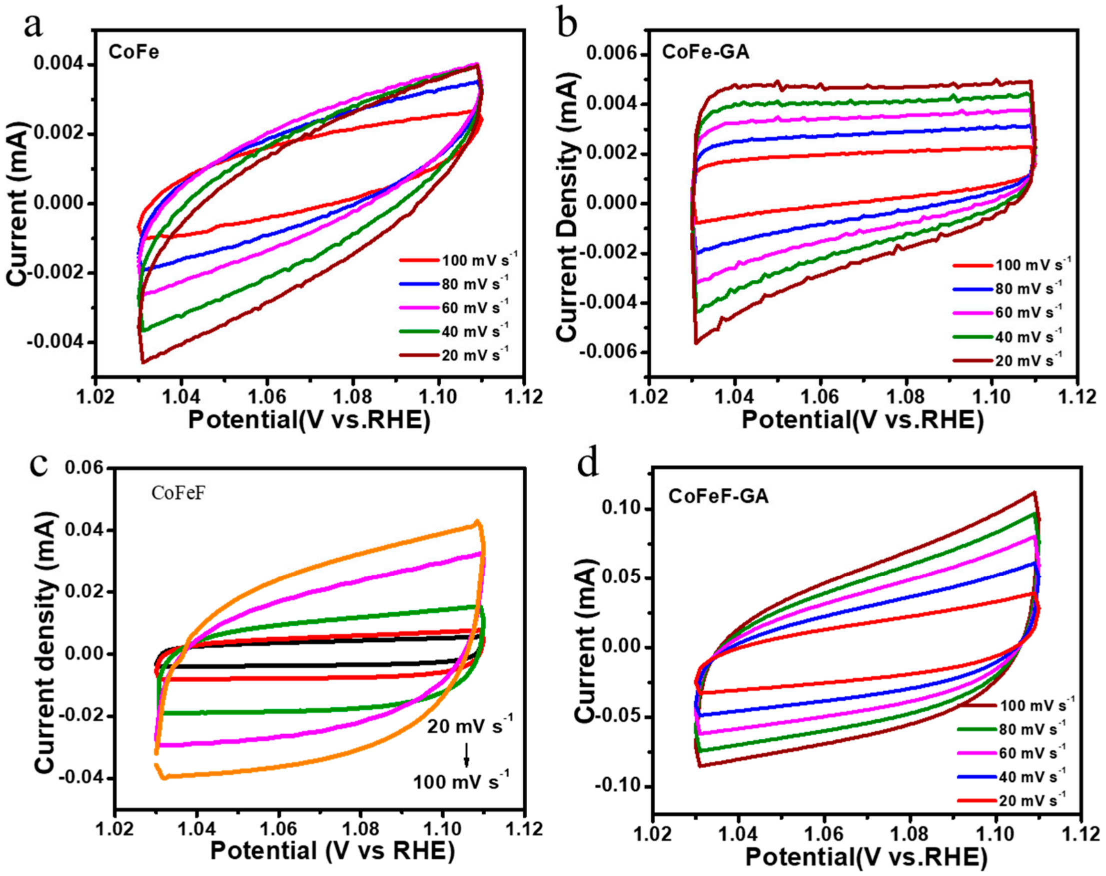

Figure S5 CV curves of (a) CoFe, (b) CoFe-GA, (c) CoFeF (d) CoFeF-GA in 1 M KOH at scan rates of 20, 40, 60, 80 and 100 mV s^{-1}.

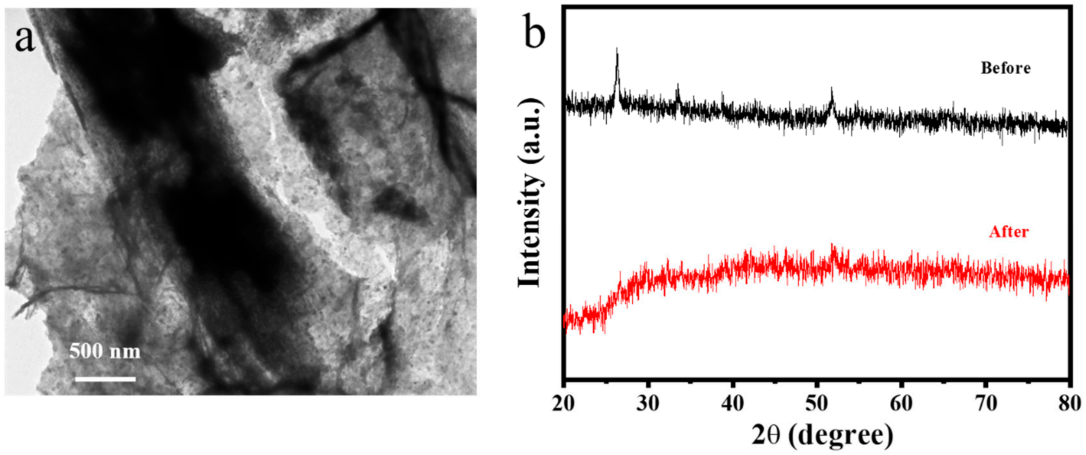

Figure S6 TEM image and XRD pattern of the CoFeF-GA catalyst after continuous CA test in 1.0 M KOH.

Table S1 The comparison of other OER catalysts derived from transition metal-based materials in 1M KOH (η: overpotential at the current density of 10 mA cm^{-2})

**Materials**	**Morphology**	**Overpotential (mV)**	**Ref**
CoFeF-GA	Nanosheets	245	This work
NiCoFe-LDH/CFC	Nanosheets	280	[1]
$KCo_{0.80}Fe_{0.20}F_3$	Nanocubics	254	[2]
$Co_{0.3}Ni_{0.3}Fe_{0.2}S$ NPs/C	Nanoparticles	266	[3]
Meso-NPC/Co_2NiO_x	Hollow spheres	330	[4]
Fe,$CoZn_{9+9}$-NO/WC	Wrinkled-nanocake	290	[5]
NiFeP–WO_x	Nanosheets- nanowires	270	[6]
Fe-Co@NSDC	Nanocages	296	[7]
$(Fe, Co, Ni)_9S_8$/NSCFs	Nanofibers	390	[8]
CNTAs-NG	Nanotubes	340	[9]
NiCoFe-LDH HP	Hollow polyhedron	276	[10]
$Co(OH)F/Ni(OH)_2@Fe(OH)_3$-D1	Core-shell	270	[11]

Table S2. The fitted Rs, Rct and R1 values of all catalysts.

Catalysts	R_s/Ω	R_{ct}/Ω	R_1/Ω
CoFeF	8.01	44.9	4.07
CoFe	11.84	160.7	3.51
CoFe-GA	10.52	144.9	18.62
CoFeF-GA	9.53	17.74	1.74

Table S3. Detailed values of Cdl, ECSA and Rf of all catalysts.

Samples	C_{dl}(mF cm^{-1})	$ECSA$(cm^{-2})	R_f
CoFe	0.01	0.0175	0.25
CoFeF	0.32	0.56	8
CoFe-GA	0.03	0.0525	0.75
CoFeF-GA	0.50	0.875	12.5

References

1. Wang T.;Xu W. and Wang H. Ternary NiCoFe Layered Double Hydroxide Nanosheets Synthesized by Cation Exchange Reaction for Oxygen Evolution Reaction. *Electrochim. Acta*. **2017**, *257*, 118-127
2. Li W.;Murisana A.;Zhang Q.;Wang S. and De G. Controlled synthesis and M-position regulation of perovskite fluoride KMF_3 (M=Co/Fe) with high-efficiency OER performance. *Electrochem. Commun.* **2022**, *141*, 107363
3. Wang G.;Cao W.;Zhang Z.;Liu L.;Wang C.;Yin J. and Zhou H. Ternary sulfide nanoparticles anchored in carbon bubble structure for oxygen evolution reaction. *J. Alloys Compd.* **2023**, *968*, 172314
4. Wang J. and Zeng H. C. Hybrid OER Electrocatalyst Combining Mesoporous Hollow Spheres of N, P-Doped Carbon with Ultrafine Co_2NiO_x. *ACS Appl. Mater. Interfaces*. **2020**, *12*, 50324-50332
5. Xue J.;Liu Z.;Fan Y.;Wang R. and Li Y. ZIF-derived Fe,Co coordinated N/O-codoped three-dimensional tungsten–carbon matrix for the performance-enhanced zinc-air flow battery and water splitting. *Chem. Eng. J.* **2023**, *476*, 146502
6. Kim D.;Jeong Y.;Roh H.;Lim C. and Yong K. Biomimetic 2D-Ni(Co,Fe)P/1D-WOx nanocoral reef electrocatalysts for efficient water splitting. *J. Mater. Chem. A*. **2021**, *9*, 10909-10920
7. Li Z.;Zhang L.;Zhu Q.;Ke Z. and Hu G. Spatial separation strategy to construct N/S co-doped carbon nanobox embedded with asymmetrically coupled Fe-Co pair-site for boosted reversible oxygen electrocatalysis. *J. Colloid Interface Sci.* **2024**, *653*, 1577-1587
8. Jiang T.;Dai P.;Zhang W. and Wu M. Fish bone-derived N, S co-doped interconnected carbon nanofibers network coupled with $(Fe, Co, Ni)_9S_8$ nanoparticles as efficient bifunctional electrocatalysts for rechargeable and flexible all-solid-state Zn-air battery. *Electrochim. Acta*. **2021**, *373*, 137903
9. Zhang Y.;Wang P.;Yang J.;Li K.;Zhang N.;Liu G.;Duan Y. and Qiu J. Mechanochemical coordination self-assembly for Cobalt-based metal-organic framework-derived bifunctional oxygen electrocatalysts. *J. Colloid Interface Sci.* **2022**, *613*, 733-746
10. Qin Y.;Wang F.;Shang J.;Iqbal M.;Han A.;Sun X.;Xu H. and Liu J. Ternary NiCoFe-layered double hydroxide hollow polyhedrons as highly efficient electrocatalysts for oxygen evolution reaction. *J. Energy Chem.* **2020**, *43*, 104-107
11. Jiu H.;Wei H.;Wang C.;Che S.;Guo Z.;Han Y.;Xu Q.;Yu X. and Zhang L. Construction of $Co(OH)F/Ni(OH)_2@Fe(OH)_3$ core-shell heterojunction on nickel foam for efficient oxygen evolution. *Int. J. Hydrogen Energy*. **2022**, *47*, 33719-33727
